# Prevalence and characteristics of overweight/obesity in children newly diagnosed with type 1 diabetes in Kuwait

**DOI:** 10.3389/fendo.2026.1769706

**Published:** 2026-04-02

**Authors:** Dalia Al-Abdulrazzaq, Maira Alsaeed, Fatima Al-Ghadban, Aisha Alsaqabi, Doaa Elharifa, Abeer Samir, Hessa Al-Kandari

**Affiliations:** 1 Department of Pediatrics, College of Medicine, Kuwait University, Safat, Kuwait; 2 Department of Population Health, Dasman Diabetes Institute, Kuwait City, Kuwait; 3 Ministry of Health, Kuwait City, Kuwait; 4 Department of Public Health Practice, College of Public Health, Kuwait University, Safat, Kuwait

**Keywords:** children, diabetes, obesity, overweight, type 1 diabetes

## Abstract

**Introduction:**

The coexistence of overweight/obesity and type 1 diabetes (T1D) may increase disease burden and management complexity. We assessed the prevalence of overweight/obesity in children newly diagnosed with T1D in Kuwait and characterized their baseline clinical, metabolic, and immunological features.

**Materials and methods:**

This retrospective review analyzed medical records of children aged 14 years or younger diagnosed with T1D from 2017 to 2022. Data were obtained from the Childhood-Onset Diabetes electronic Registry (CODeR). Overweight/obesity was defined using WHO growth standards, and T1D diagnosis was confirmed based on the 2018 ISPAD guidelines.

**Results:**

From 2,066 children with T1D in CODeR, 1,265 had BMI data and were included in the present study. Of these, 29.9% (378 children) were overweight/obese at diagnosis. Males were more likely to be overweight/obese than females (55.3% vs. 44.9%, p=0.001). Overweight/obese children were older at diagnosis compared to non-obese children (median age 9.3 vs. 7.3 years, respectively, p=0.0001). These children had a milder clinical presentation, with fewer cases of DKA, lower HbA1c, and lower triglycerides at diagnosis. After adjusting for age and gender, higher BMI z-scores were associated with lower HbA1c levels, reduced odds of DKA, and lower odds of celiac autoimmunity.

**Conclusion:**

Overweight/obesity influence the presentation of type 1 diabetes in children, highlighting the need for early screening and targeted prevention. Longitudinal follow-up with genetic integration is warranted to better understand their relationship with disease progression and metabolic risk.

## Introduction

The global prevalence of childhood overweight/obesity has seen a significant rise in recent years (1). According to the UNICEF’s estimates, in the year 2022 around 37 million children under the age of 5 years are overweight or obese (2). Being overweight/obese in childhood is associated with an earlier onset of chronic diseases, which can adversely impact quality of life, educational attainment, and lead to various psychosocial challenges (3). While childhood overweight/obesity alone is associated with such numerous challenges, the coexistence of type 1 diabetes (T1D) further amplifies the disease burden, complicates management, and increases the risk of long-term health consequences. Like overweight/obesity, the rising prevalence and incidence of T1D in children has become a growing global concern (4). According to the 10th edition of the Diabetes Atlas, 1.52 million individuals (17%) under the age of 20 were living with T1D in 2022 (5). Particularly concerning is Kuwait’s ranking as the third highest among countries with the highest age-standardized incidence of T1D in children aged 0-14, with an incidence rate of 41.7 per 100,000 per year (6, 7). This has become an increasing concern to the country as incidence rate has doubled in the past two decades compared to the 1990s (6).

As the incidence of overweight/obesity and diabetes continues to rise, more children are expected to be diagnosed with both T1D and overweight/obesity. Therefore, it is imperative that the association between overweight/obesity in children diagnosed with T1D be thoroughly explored. Various studies have reported a high prevalence of overweight/obesity among children and adolescents with T1D (8–10). Moreover, an increasing prevalence of overweight/obesity has been observed alongside metabolic syndrome, a serious predictor of cardiovascular risk, specifically among children and adolescents with T1D compared to healthy controls (8, 11, 12). Overweight/obesity may exacerbate insulin resistance and contribute to metabolic complications in such population. Understanding this complex relationship between overweight/obesity and T1D in children and adolescents is crucial for providing optimal clinical management and developing targeted interventions and comprehensive strategies to address this growing health issue.

The aim of this study was to report on the prevalence of overweight/obesity in children newly diagnosed with T1D in Kuwait, as well as describe their baseline clinical, metabolic, and immunological characteristics at the time of diagnosis.

## Materials and methods

This was a retrospective review of medical records of children aged 14 years or younger at the time of diagnosis with T1D during the period from 2017 till 2022. Children were registered at the Childhood-Onset Diabetes electronic Registry (CODeR) from all six secondary diabetes centers across the state of Kuwait. Only children with documented weight and height measurements at diagnosis were included, enabling accurate BMI calculation. Patients with missing BMI data, most commonly due to absence of recorded height at admission, were excluded from the analysis.

The information collected from the medical records included demographic data, anthropometric measures (weight and height, used to calculate body mass index (BMI) as per the WHO growth standards (13)), family history of cardiovascular disease risk factors including diabetes, hypertension, dyslipidemia, and presentation with diabetic ketoacidosis (DKA). The International Society of Pediatric and Adolescent Diabetes guidelines of 2018 were followed to confirm the diagnosis of T1D and define DKA and its severity (14). Overweight/obesity was defined according to the WHO growth standards (13). As per the WHO growth standards, children aged 5 and younger, overweight was defined as BMI z-scores of more than +2, whereas obesity was defined as BMI z-scores of more than +3. In children older than 5 years of age, overweight was defined as a BMI z-score of above +1, and obesity was defined as a BMI z-score above +2 (13).

Information on biochemical and immunological investigations were collected including Hemoglobin A1C (HbA1C) expressed in (%), Triglycerides (TG) expressed in mmol/L, C-peptide expressed in pmol/L with a reference range of 160-1100, Vitamin D levels (25-OH-Vitamin D) expressed in nmol/L with a reference range of 75-125, anti-Glutamic acid decarboxylase (GAD) antibodies, thyroid antibodies, and celiac antibodies at the time of diagnosis.

Pancreatic autoantibodies were reported as part of routine clinical care at diagnosis across participating centers. Testing was performed in local laboratories using assays available at the time, without centralized standardization of platforms or cut-off values. The CODeR registry captures autoantibody status as reported clinically (positive/negative). Due to temporal changes in assay reagent availability, not all patients had a complete pancreatic autoantibody profile. GAD antibodies were the most consistently available across the cohort and were therefore reported in the present study. A positive thyroid antibody status was defined when thyroid peroxidase antibodies (anti-TPO) and/or Thyroglobulin antibodies (anti-TG) were positive. Whereas a positive celiac antibody status was defined when Tissue Transglutaminase antibodies (anti-TTG) and/or Endomyseal antibodies (EMA) were positive.

Data was analyzed using Stata 17.0 software. Continuous variables were expressed as mean ± Standard Deviation (SD) if normally distributed or median (Interquartile range-IQR) if not normally distributed. Categorical variables were reported as counts and percentages. As BMI was the primary exposure of interest, complete cases analysis was performed. Missing BMI values were not imputed, and no sensitivity analyses were performed, to avoid potential misclassification and biased prevalence estimates. The equality of two means or two medians was tested using the two-sample test if the data showed normality; otherwise, the Mann-Whitney U test was used. To test the association between categorical variables, the Pearson chi-square test of independence was implemented. To model the association between clinical and biochemical outcomes, namely HbA1c, presence of DKA, thyroid autoimmunity, and celiac autoimmunity with BMI z-score and possible confounding covariates, linear and logistic regression modeling was implemented. Assumptions of linear regression models were assessed using graphical plots (not presented). All tests were two-tailed, and a significant level was set at 5%.

The study was performed in accordance with the Declaration of Helsinki and was approved by the Standing Committee for Coordination of Health and Medical Research at the Ministry of Health [RA-2011-006].

## Results

**Figure 1:**
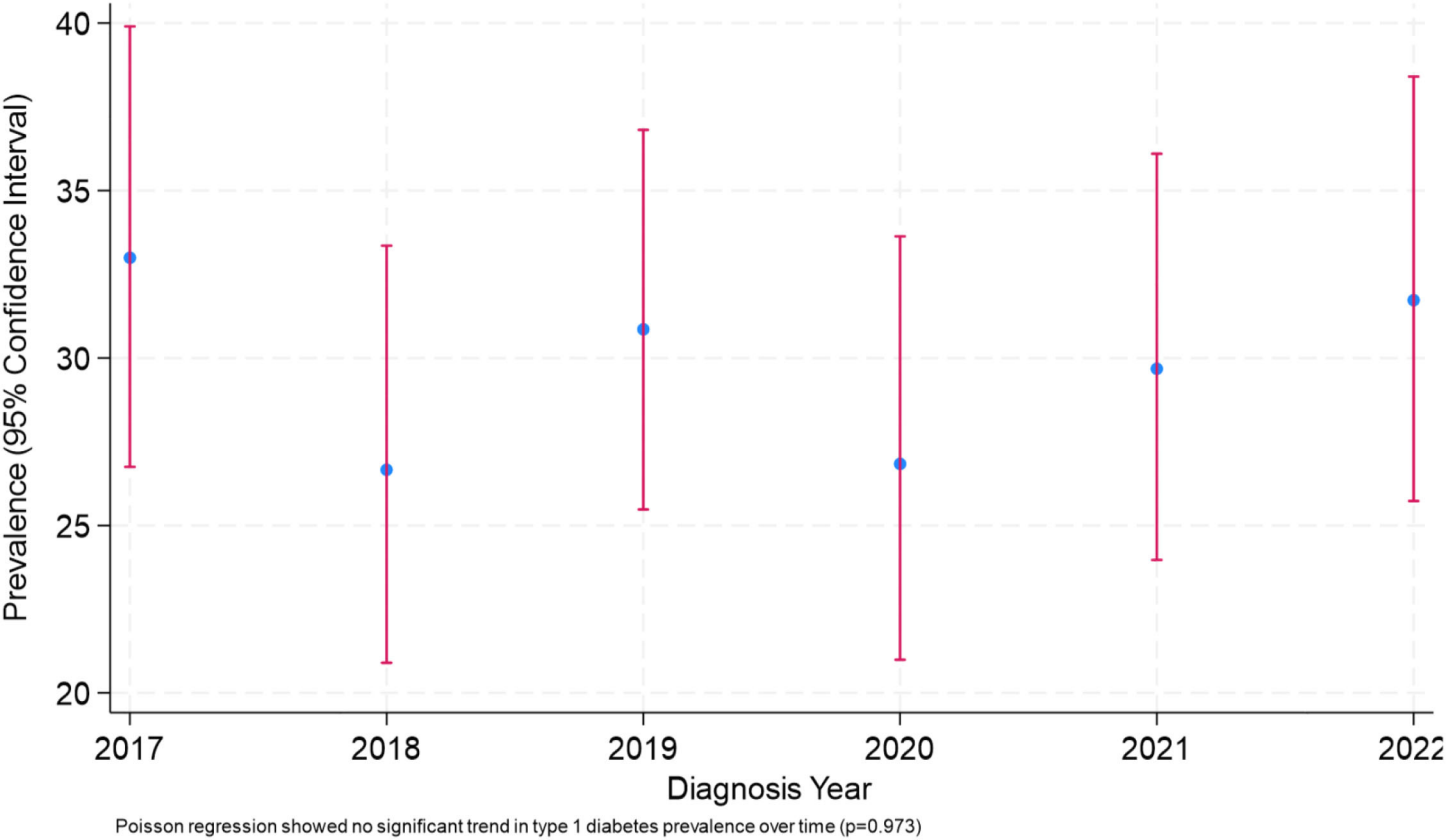
Descriptive annual prevalence of overweight/obesity among children at type 1 diabetes diagnosis (2017–2022).

During the study period, 2066 children were registered to have T1D in CODeR of whom 1265 (61.2%) children had information on BMI at the time of diagnosis and therefore were included in the present study. Baseline characteristics of the study cohort with comparison between overweight/obese and non-obese children are demonstrated in **Table 1**. Almost a third of the study population (378, 29.9%) were overweight/obese at the time of diagnosis with T1D. Between 2017 and 2022, there was no significant change in the percentage of overweight/obese children over the individual study years (**Figure 1**).

**Table 1:** Baseline characteristics of children with overweight/obesity and were newly diagnosed with type 1 diabetes.

Variable	Total N=1265 (100.0%)	Overweight and obese N= 378 (29.9%)	Non-Obese N= 887 (70.1%)	P-value
Male gender, n (%)	607 (48.0)	209 (55.3)	398 (44.9)	0.001
Kuwaiti national, n (%)	903 (71.4)	284 (75.1)	619 (69.8)	0.054
Age in years, median (IQR)	8.0 (5.3, 10.1)	9.3 (7.3, 10.7)	7.3 (4.4, 9.4)	0.001
BMI z-score, mean (SD)	0.20 (1.94)	2.42 (1.05)	-0.74 (1.39)	N.A
Family history of CVS risk factors, n (%)	535 (42.3)	93 (44.5)	170 (42.7)	0.673
DKA, n (%)	492 (41.1)	122 (34.1)	370 (44.1)	0.001
Severe DKA, n (%)	127/492 (25.8)	29 (23.8)	98 (26.5)	0.518
HbA1c%, mean (SD)	11.2 (2.1)	10.9 (1.9)	11.3 (2.2)	0.001
TG in mmol/L, (median (IQR)*	1.27 (0.81, 2.31)	1.15 (0.78, 1.96)	1.37 (0.83, 2.5)	0.001
C-peptide^a^ in pmol/L, median (IQR)*	125.0 (71.0, 236.0)	237.7 (135.0, 450.0)	99.0 (59.0, 167.0)	<0.001
Vitamin D^a^ in nmol/L, median (IQR)*	40.0 (27.0, 55.0)	33.8 (22.3, 44.2)	43.6 (29.2, 58.8)	<0.001
Positive GAD^b^, n (%)	541/723 (74.8)	169 (77.2)	372 (73.8)	0.339
Thyroid autoimmunity, n (%)	137/712 (19.2)	36 (15.6)	101 (21.0)	0.086
Celiac autoimmunity, n (%)	93/1,027 (9.1)	22 (7.1)	71 (9.9)	0.139

BMI, body mass index; IQR, interquartile range; CVS risk, cardiovascular system risk (family history of diabetes mellitus and hypertension); DKA, diabetic ketoacidosis; HbA1c, haemoglobin A1c; TG, triglyceride; GAD, anti–glutamic acid decarboxylase antibody; Anti-TPO, anti–thyroid peroxidase antibody; Anti-TG, anti–thyroglobulin antibody; Anti-TTG, anti–tissue transglutaminase antibody; EMA, anti–endomysial antibody.

*Total might not add up due to some missing data.

a Clinical Reference Ranges: C-peptide 160–1100 pmol/L; Vitamin D levels (25-OH-Vitamin D) 75–125 nmol/L.

b Total might not add up due to some missing data.

c Either anti-TPO and/or anti-TG is positive.

d Either anti‐TTG and/or EMA is positive.

Male children were more likely to be overweight/obese at the time of diagnosis with T1D than girls (55.3% vs. 44.9%, p=0.001). Children with overweight/obesity were older at the time of diagnosis compared to non-obese children (median age 9.3 and 7.3 years, respectively, p=0.0001). Furthermore, children with overweight/obesity had a milder clinical presentation at the time of diagnosis with less children presenting with DKA, lower HbA1C (%), and lower TG (mmol/L) at diagnosis (**Table 1**). Moreover, they had a higher C-peptide level at diagnosis (Median 237.7 pmol/L vs 99.0 pmol/L, p=0.0001). Vitamin D levels (25-OH-Vitamin D) were lower (33.8 nmol/L vs 43.6 nmol/L, p=0.0001) in the group of children with overweight/obesity children. Prior to adjustment, thyroid autoimmunity was slightly lower in children with overweight/obesity children compared to non-obese children with no difference in the positivity of anti-GAD antibodies and celiac antibodies (**Table 1**).

In **Table 2**, a logistic regression model was used to test for a possible association between HbA1C (%), DKA, thyroid and celiac autoimmunity, and BMI z-scores (accounting for gender and age) at the time of diagnosis. Higher BMI z-scores at diagnosis were significantly associated with lower HbA1c levels (β = -0.25; 95% CI: -0.30, -0.19; p<0.0001). Higher BMI z-scores were also associated with reduced odds of DKA at presentation (OR 0.88; 95% CI: 0.83, 0.94; p<0.0001) and lower odds of celiac autoimmunity (OR 0.85; 95% CI: 0.76, 0.95; p=0.004). The association between BMI z-scores and thyroid autoimmunity was not statistically significant (OR 0.92; 95% CI: 0.82, 1.02; p=0.097). Multicollinearity was assessed in the fitted regression model, and the variance inflation factor was 1.02, indicating no evidence of collinearity among the covariates.

**Table 2:** Outcomes associated with body mass index (BMI) z-scores in children newly diagnosed with type 1 diabetes in Kuwait.

Outcomes	Effect estimate	95% Confidance interval	P-value
HbA1C	N= 1,229		
Variable	β Coefficient	95% Confidence interval	P-value
Male gender	-0.41	-0.63, -0.19	0.0001
Age in years	0.21	0.17, 0.25	0.0001
BMI z-scores	-0.25	-0.30, -0.19	0.0001
DKA	N=1,196		
Variable	OR	95% Confidence interval	P-value
Male gender	0.80	0.63, 1.01	0.064
Age in years	0.98	0.94, 1.017	0.262
BMI z-scores	0.88	0.83, 0.94	<0.0001
Thyroid autoimmunity^a^	N=712		
Variable	OR	95% Confidence interval	P-value
Male gender	0.52	0.35, 0.78	0.001
Age in years	1.01	0.95, 1.08	0.752
BMI z-scores	0.92	0.82, 1.02	0.097
Celiac autoimmunity^b^	N=1,027		
Variable	OR	95% Confidence interval	P-value
Male gender	0.87	0.56, 1.34	0.519
Age in years	1.09	1.01, 1.17	0.029
BMI z-scores	0.85	0.76, 0.95	0.004

HbA1c, haemoglobin A1c; BMI, body mass index; DKA, diabetic ketoacidosis; Anti-TPO, anti–thyroid peroxidase antibody; Anti-TG, anti–thyroglobulin antibody; Anti-TTG, anti–tissue transglutaminase antibody; EMA, anti–endomysial antibody; OR, odds ratio.

a Either anti-TPO and/or anti-TG is positive.

b Either anti‐TTG and/or EMA is positive.

## Discussion

This study examined the prevalence of overweight/obesity among children newly diagnosed with T1D in Kuwait, alongside their baseline clinical, metabolic, and immunological characteristics. Among the children with complete data on BMI at diagnosis, nearly one-third were classified as overweight/obese at diagnosis. After adjusting for age and gender, higher BMI z-scores were associated with lower HbA1c (%) levels, reduced odds of DKA, and lower odds of celiac autoimmunity.

The prevalence of overweight/obesity among the study population aligns with global findings, highlighting the concerning trend regarding pediatric overweight/obesity in the general and T1D populations. A comprehensive analysis utilizing data from the Kuwait Nutrition Surveillance System (KNSS) for children aged 5–19 years over a 13-year period (2007–2019) revealed a significant increase in overweight/obesity rates, with a rate of 20.2% and 28.4% respectively in 2019 (15). Using the same criteria for defining overweight/obesity in children, the international SWEET registry, which included over 23,000 children and adolescents with T1D from multiple centers worldwide, had reported a similar prevalence of overweight/obesity with a rate of 31.8% (9). Similarly, the T1D Exchange Clinic Registry in the United States reported a prevalence of 34.7% of overweight/obesity in participants with T1D (16), whereas a study from Turkey reported a lower prevalence of overweight/obesity (18.0%) in children aged 8–18 years with T1D in comparison to our study (17). These international findings suggest that the prevalence of overweight/obesity in children with T1D is comparable or exceeding the prevalence of overweight/obesity in the general population. However, in our study, the prevalence of overweight/obesity in children with T1D was lower in comparison to the general population. Due to the cross-sectional nature of the current study, whether the prevalence of overweight/obesity in children with T1D a reflection of the rising overweight/obesity epidemic nationally and globally or a potential role of overweight/obesity in the development of T1D in genetically susceptible children. A systematic review and meta-analysis by Nitecki et al. found a significant association between high BMI and increased risk of development of T1D, reporting a pooled relative risk of 1.87 for overweight/obese individuals compared to those with normal weight (18). A study by March et al. observed that the prevalence of overweight/obesity had increased threefold at the time of diagnosis of T1D over two decades, indicating a shift in the clinical presentation of the disease (19). Nevertheless, the percentage of children with overweight/obesity in our study remained consistent over the five-year period, from 2017-2022, suggesting that the issue is persistent and warrants targeted and early public health interventional strategies.

Further aligning with overweight/obesity trends observed in in the general pediatric population in Kuwait and other regions, our study found that male children with T1D were more likely to be overweight/obese at diagnosis of T1D. Al-Taiar et al. documented an increasing prevalence of obesity among Kuwaiti males, whereas obesity rates among females remained stable (15). However, there has been different evidence on gender difference in children with obesity and T1D. Mirroring our findings, Bitkin et al. reported that male children with T1D had higher BMI z-scores at diagnosis (20). Whereas the international SWEET registry had reported that the BMI z-scores were significantly higher in females 2018 (9). Age-related differences in overweight/obesity prevalence have been well documented in the literature as well. Our findings, which indicate that older children are more likely to be overweight/obese at T1D diagnosis, are consistent with trend from the general population in Kuwait (15). With regards to children with T1D, the international SWEET registry reports a difference in BMI z-scores by age which is further influenced by gender where in males the BMI z-scores decreased by age whereas it had a U-shaped pattern in females where it was higher in youngest and eldest age groups (9). A study conducted in Australia had reported on increased rates of overweight/obesity by age across both sexes (21). Additionally, Fröhlich-Reiterer et al. found that older children with T1D exhibited higher BMI and were more likely to develop overweight/obesity over time (22). While it appears that overweight/obesity play a role in T1D, the specific age at which it does remains unclear due to variations in study designs, age assessment, and the studied populations. Nevertheless, our findings highlight the need for age- and gender-specific preventive strategies to be implemented at the national level.

However, it should be noted that comparisons with international registries and studies should be interpreted with caution, as differences in age structure, diagnostic practices, population characteristics, and definitions of overweight/obesity may influence reported prevalence and associations. Furthermore, variations in background overweight/obesity rates and healthcare systems may also contribute to the observed differences.

From a clinical and biochemical perspective, overweight/obese children in our cohort presented with lower DKA rates and had higher residual beta-cell function, as indicated by higher C-peptide (pmol/L) levels at diagnosis as well as lower HbA1C (%) and triglyceride (mmol/L) levels. These findings are consistent with those of Bitkin et al. and Fröhlich-Reiterer et al., who report that excess weight at diagnosis may be associated with less severe metabolic decompensation (20, 22). These findings support the “accelerator hypothesis” which has been generalized on the Arab population of children newly diagnosed with T1D in Kuwait (23). The “accelerator hypothesis” proposes that increased adiposity may enhance insulin resistance and modify the clinical expression of T1D in susceptible individuals (24). In populations such as Kuwait, where childhood overweight/obesity prevalence is high and genetic susceptibility to diabetes is well recognized, the interaction between excess adiposity and underlying autoimmune predisposition may contribute to the heterogeneity observed at diagnosis where an increasing number of overweight/obese children with pancreatic autoantibodies exhibit features of insulin resistance, a phenotype often described as “double diabetes” (25). This overlap has been reported more frequently in regions undergoing rapid nutritional and lifestyle transitions, including Middle Eastern countries with high pediatric overweight/obesity rates (26). However, given the cross-sectional design of the present study, these observations should be interpreted as hypothesis-generating rather than causal. Furthermore, alternative explanations should also be considered like differences in healthcare access, parental symptom recognition, timing of presentation, and diagnostic delay which may contribute to variations in metabolic severity. In addition, sociocultural and healthcare system factors within the region may influence patterns of presentation independent of biological mechanisms. Therefore, the observed associations between BMI and disease characteristics at diagnosis should be interpreted within the broader context of both biological and healthcare-related determinants.

Our study reports on a less likelihood of developing celiac autoimmunity with children with higher BMI z-scores. This finding aligns with those of Bitkin et al., who also noted similar clinical profiles in overweight/obese children at T1D diagnosis, suggesting that higher BMI may be protective against autoimmune comorbidities in T1D, although the underlying mechanisms remain unclear (20). A study by Kaspers et al. and another by Craig et al. found that children with T1D and higher BMI were less likely to develop celiac disease, potentially due to differences in immune system activation or genetic predisposition (27). Research had demonstrated that overweight/obese youth with newly diagnosed T1D may have altered pro-inflammatory profile including higher levels of leptin, visfatin, chemerin, TNF-α, and CRP, and lower levels of adiponectin and omentin which in turn might influence the overall immune response leading to the development of T1D (19). Although our study did not report on differences with regards to overweight/obesity and pancreatic autoimmunity namely GAD antibodies, studies have shown that adiposity accelerates islet autoimmunity prior to the clinical presentation of T1D (28). However, it should be noted that in the current study, information on GAD antibodies only was available for comparison between the studied group. The immunological profile in children with overweight/obesity and T1D has been a subject of research, and further studies are needed to explore these associations and their clinical implications.

Overall, our findings align with global trends while also offering unique insights into the pediatric T1D population in Kuwait. A key strength of this study is its reliance on the Childhood Onset Diabetes Registry (CODeR), a comprehensive, national electronic registry that systematically collects data on children diagnosed with diabetes in Kuwait. The well-established nature of this registry ensures robust data collection and enhances the representativeness of the study population, providing a reliable snapshot of pediatric T1D cases in Kuwait. However, several limitations must be acknowledged. First, the cross-sectional nature of the study limits our ability to assess longitudinal changes in weight or clinical characteristics and precludes causal inference; therefore, overweight/obesity cannot be interpreted as a risk factor for the development or onset of T1D and its related metabolic complications, but rather as a characteristic observed at the time of diagnosis. A limitation of this study as well is that BMI data were available for approximately 61% of eligible patients, reflecting incomplete anthropometric documentation at diagnosis. However, inclusion was restricted to patients with objectively recorded height and weight to ensure accurate BMI classification. Pancreatic autoantibody testing was not standardized across centers, reflecting real-world registry-based practice. Variability in assays, changes in laboratory platforms over time, and incomplete testing, particularly during the COVID-19 period, led to missing full pancreatic autoantibody data, limiting evaluation of the relationship between adiposity and islet autoimmunity at diagnosis. Moreover, the CODeR registry lacked information on birth and neonatal factors, as well as puberty status and family history of other autoimmune disease which might be key determinants in the interplay between overweight/obesity and T1D, underscoring a critical gap and an opportunity for future research, particularly in the context of preventive strategies. The absence of socioeconomic data also limits our ability to explore potential disparities or associations related to social determinants of health. Including such information in future studies would provide a more comprehensive understanding of the interplay between overweight/obesity and T1D in children. Finally, although registry-based data may carry a small risk of coding or entry errors, the CODeR registry incorporates rigorous multi-stage data verification processes to enhance data accuracy and reliability.

## Conclusion

The findings of this study have important public health and clinical implications, particularly in Kuwait and the wider Middle Eastern region, where childhood obesity remains highly prevalent. The co-occurrence of overweight and obesity with T1D at diagnosis reflects the evolving clinical phenotype of pediatric diabetes and its potential long-term metabolic consequences.

Given the substantial regional burden of overweight/obesity and diabetes, and the distinct genetic background of Middle Eastern populations, further research is warranted to better understand the interaction between clinical risk factors and genetic susceptibility in shaping disease presentation and progression. Longitudinal follow-up through linkage with the CODeR registry will be essential to determine how overweight/obesity at diagnosis may relate to subsequent clinical outcomes and disease trajectory over time.

Our findings highlight the critical need for early detection and intervention. Comprehensive public health strategies are essential to address the complex relationship between overweight and obesity and T1D in children and adolescents, emphasizing early screening, lifestyle modifications, tailored interventions, and targeted management approaches to address obesity and its associated metabolic risks among children with T1D.

## Data Availability

Data are available from the author(s) with the permission of Dasman Diabetes Institute and Ministry of Health of Kuwait.
